# Advances in Crosstalk Reduction Techniques for Ultrasonic Transducer Arrays

**DOI:** 10.3390/s25247666

**Published:** 2025-12-18

**Authors:** Anouar Boujenoui, Nadia El Atlas, Abdelmajid Bybi, Hayat Reskal, Lahoucine Elmaimouni

**Affiliations:** 1 MEAT—Materials Energy and Acoustics Team, Higher School of Technology in Salé, Mohammed V University in Rabat, Rabat 10100, Morocco; nadiaelatlas@gmail.com (N.E.A.); abdelmajid_bybi@hotmail.fr (A.B.); reskal.hayat@gmail.com (H.R.); 2 Laboratory Physics, Energy and Information Processing-Renewable Energy, Acoustic and Mechanical Microsystems, Polydisciplinary Faculty of Ouarzazate, Ibn Zohr University, BP 638, Ouarzazate 45000, Morocco; la_elmaimouni@yahoo.fr

**Keywords:** ultrasonic transducer, crosstalk, cross-coupling, array transducer

## Abstract

Crosstalk between elements in ultrasonic transducer arrays significantly degrades image quality in medical ultrasound systems by introducing noise and reducing spatial resolution. This review provides a comprehensive overview of the origins of crosstalk—acoustic, mechanical, and electrical—and the main characterization methods used to analyze it, including direct measurements, impedance analysis, finite element modeling, and equivalent circuit approaches. Emphasis is placed on recent advances in passive and active mitigation strategies, such as material coatings, structural decoupling, phononic crystals, adaptive filtering, and impedance matching. A key finding is that the optimal crosstalk reduction method depends strongly on the transducer technology employed—whether CMUT, PMUT, or bulk PZT. The review highlights the importance of tailoring mitigation techniques to the physical properties and operating conditions of each technology. By synthesizing current knowledge and identifying remaining challenges—particularly the role of filler material losses—this work offers a solid foundation for the development of next-generation ultrasound arrays with enhanced imaging performance.

## 1. Introduction

The design and fabrication of ultrasonic transducer arrays for medical imaging face multiple critical challenges that impact device performance and imaging quality. Achieving fine structural features and high electromechanical coupling coefficients is essential to ensure efficient conversion between electrical and acoustic signals, especially at high frequencies above 20 MHz [1,2]. Material selection and multilayer stack optimization—including backing absorbers and matching layers—are key factors to manage acoustic impedance mismatch and enhance bandwidth [3,4,5]. Additionally, fabrication limitations, such as precise membrane sizing, stress management, and integration complexities, influence the array uniformity and durability, particularly for MEMS-based technologies like CMUTs and PMUTs [2,6,7]. The increasing miniaturization and dense packing of elements to improve spatial resolution pose challenges related to wiring complexity and electrical interference [8,9].

Among the major challenges facing the design and fabrication of ultrasound transducer arrays, inter-element crosstalk remains a critical limitation that directly affects imaging performance. Recent advances in flexible and conformable ultrasound arrays have further highlighted the critical role of crosstalk mitigation in modern imaging systems. For instance, van Neer et al. (2024) introduced large-area polymer-based ultrasound arrays designed for conformable medical applications [10]. Similarly, Elloian et al. (2022) reported a 256-element flexible piezoelectric array with geometric phase correction, which significantly reduces inter-element coupling on curved surfaces [11]. These developments underscore that reducing crosstalk is not only essential for conventional rigid arrays but also for emerging flexible and wearable devices. Crosstalk leads to degraded beam formation, resulting in poor spatial resolution and contrast, particularly in diagnostic applications where image clarity is essential for accurate interpretation [12]. It often arises from spurious vibration modes that propagate through the backing material and kerf filler, coupling neighboring elements and distorting the radiated field—especially at large steering angles [13]. In MEMS-based technologies such as CMUTs, the low-loss silicon substrate exacerbates this phenomenon due to its high acoustic conductivity and continuous structure [12]. To address these effects, several mitigation strategies have been proposed. Programmable waveform techniques, which modify transmit signals on adjacent elements, can reduce acoustic crosstalk by up to 25 dB without requiring hardware changes [14]. Structural interventions such as etched isolation trenches, damping materials, and polymer walls between elements have proven effective in disrupting unwanted propagation paths [15]. Additionally, material innovations—like the use of pseudo-random 1–3 piezocomposites—suppress interpillar resonances at high frequencies [16]. Optimized kerf design in piezoelectric arrays also plays a crucial role in minimizing mechanical coupling and enhancing acoustic performance [17]. These approaches are central to ongoing research focused on improving array fidelity, especially as transducer technologies evolve toward higher frequencies, finer resolutions, and increased element counts.

This systematic review aims to provide a comprehensive overview of crosstalk reduction methods tailored to different ultrasonic transducer array technologies, guiding researchers and manufacturers in selecting optimal strategies that balance imaging performance and crosstalk mitigation. Several studies have highlighted the significant influence of fabrication technology on the severity of inter-element crosstalk. For instance, micro-machining techniques utilizing diaphragm structures with small windows have been shown to enhance element isolation and reduce crosstalk compared to solid substrates [18,19]. Material and structural choices, such as the incorporation of lossy filler materials or specialized grounding methods, further impact crosstalk levels by attenuating parasitic acoustic and electrical couplings, albeit sometimes at the cost of sensitivity [4,20]. Additionally, distinct transducer types require specific approaches; piezoelectric micromachined ultrasonic transducers (PMUTs) benefit from optimized electrode designs to achieve dual-mode resonances with minimized crosstalk [21], while capacitive micromachined ultrasonic transducers (CMUTs) can effectively reduce acoustic crosstalk through programmable waveform techniques combined with physical separation [14]. Numerical simulations and experimental validations complement these advances by elucidating crosstalk mechanisms and confirming the efficacy of mitigation strategies [22]. Understanding these technology-dependent effects is essential to develop tailored solutions that optimize both device sensitivity and crosstalk suppression.

In this review, we first provide a detailed overview of the crosstalk phenomenon in ultrasonic transducer arrays, including its definitions, physical mechanisms, and typologies (Section 2). We then examine how different array technologies—such as CMUTs, PMUTs, and bulk piezoelectric arrays—influence the nature and severity of crosstalk (Section 3). Subsequently, we present the principal experimental and modeling techniques used to characterize crosstalk, highlighting technology-specific constraints (Section 4). Following this, various structural, material, circuit-level, and signal processing strategies for crosstalk reduction are reviewed and classified according to the transducer technology (Section 5). Finally, we discuss and compare the effectiveness and complexity trade-offs of these approaches, identify open challenges, and suggest future research directions, before concluding the review (Section 6).

## 2. Crosstalk Phenomenon in Ultrasonic Transducer Arrays

### 2.1. Definition and Relevance

Crosstalk in ultrasonic transducer arrays is defined as the undesired coupling of energy between adjacent elements, resulting in parasitic mechanical displacements and electrical voltages that degrade the directivity and overall performance of the array [23,24]. The main causes of crosstalk include mechanical vibrations and electrical coupling transmitted through insufficient isolation between elements [25,26]. Design factors such as front matching layers, backing absorbers, and filler materials significantly influence crosstalk levels; for example, lossy fillers can reduce parasitic modes but may also compromise sensitivity [4,27].

Crosstalk impacts array performance by causing interference and distortions, which lead to reduced image quality in medical ultrasound applications [4,28]. It can introduce measurement errors that distort tomographic reconstructions, thereby affecting diagnostic accuracy [26,28]. Additionally, crosstalk restricts the effective aperture and steering capabilities of arrays, limiting their operational range in various uses [29].

### 2.2. Physical Mechanisms

The performance of ultrasound transducer arrays is influenced by three main coupling mechanisms: acoustic, mechanical, and electrical. These couplings can interact and lead to undesired effects such as crosstalk, but they also offer potential for functional innovations.

Acoustic coupling occurs when sound waves propagate between adjacent elements, often through shared media or cavities. This is particularly relevant in PMUTs, where inter-cavity coupling can cause secondary peaks in the frequency response [22]. Yet, such coupling can also be harnessed for remote excitation or frequency filtering [30].

Mechanical coupling involves the transmission of vibrations through the structure of the array, potentially distorting the acoustic field. It is a key source of crosstalk in linear medical arrays [25,31]. Proper impedance matching helps mitigate these effects, especially in high-power systems like ultrasonic welding [32].

Electrical coupling results from unintended interactions between signal lines or shared electrical paths. It can be modeled using equivalent circuit networks integrating the piezoelectric and mechanical domains [33]. Some designs separate the electrical and acoustic modules to reduce interference [34].

The interplay of these couplings is complex but essential to understand for accurate modeling and system optimization. In ultrasonic power transfer systems, for instance, the equivalence between mechanical and electrical parameters is exploited to improve efficiency [35,36]. While typically viewed as parasitic, these couplings can be leveraged for advanced functionalities and innovative system designs.

**Figure 1:**
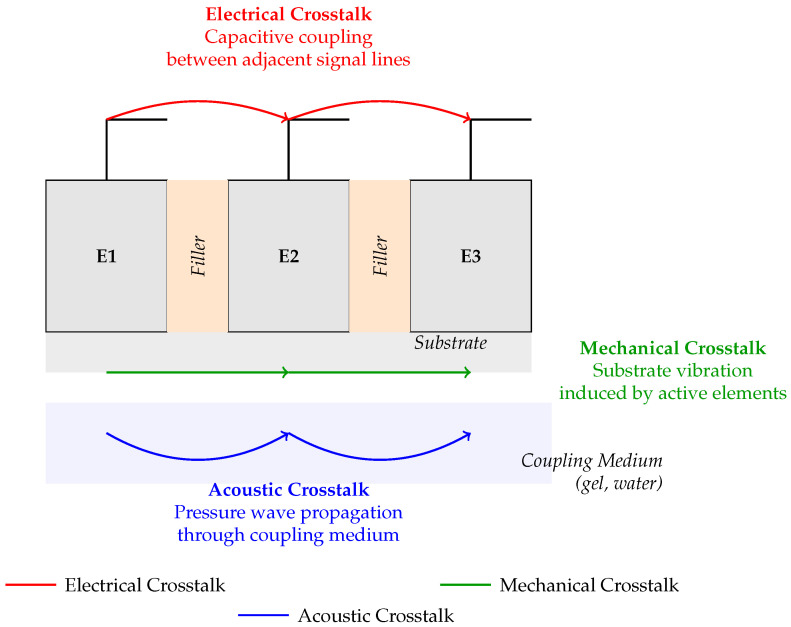
Illustration of the main types of crosstalk in ultrasonic transducer arrays.

As illustrated in Figure 1, these three types of coupling often coexist and interact within a transducer array, influencing performance and enabling novel configurations when properly controlled.

### 2.3. Crosstalk in Acoustic Levitation and Particle Manipulation

Beyond conventional ultrasonic arrays used in imaging and sensing, crosstalk also plays a critical role in advanced applications such as acoustic levitation and particle manipulation, where it directly impacts trapping stability and control accuracy. In these systems, sound waves are employed to suspend and control particles without physical contact, offering significant advantages in contamination-free environments such as extraterrestrial material analysis or microgravity studies. However, crosstalk introduces unique challenges that compromise stability and precision. Secondary acoustic forces, arising from sound scattering between levitated particles, complicate accurate control since they are difficult to measure with conventional methods; recent high-resolution techniques now provide better insights into these interactions [37]. Similarly, radial perturbations in single-axis levitators reduce axial stiffness and stability, highlighting the need to understand radial–axial coupling for improved levitator designs [38]. Advanced methods, such as higher-order transverse modes in multi-element ultrasonic cavities, have enabled simultaneous trapping of multiple particles with enhanced positional stability, thereby mitigating crosstalk through more precise control of the acoustic field [39]. Unilateral levitation using Bessel beams has further expanded manipulation capabilities by minimizing interference from multiple sources, reducing the likelihood of acoustic crosstalk [40]. Hybrid approaches like sonomaglev, which combines acoustic and diamagnetic levitation, provide stable manipulation of droplets and controlled coalescence by exploiting complementary field properties [41]. Beyond purely acoustic effects, crosstalk in levitation systems can also be electrical, due to insufficient isolation between components in GHz bulk acoustic wave (BAW) arrays [42], mechanical, through physical coupling of transducers [42], or optical, when optical tweezers are integrated with acoustic manipulation [43]. These forms of crosstalk affect the stability and accuracy of particle control, necessitating isolation strategies, advanced modeling, and innovative approaches such as phase optimization, pulse-train transitions, or simulation-guided designs [42,43,44]. While crosstalk poses limitations, it also opens opportunities for developing more sophisticated levitation systems with robust stability and high precision, which are critical for emerging biomedical, industrial, and space applications [38,39,40,41,42,43,44].

In addition to the general crosstalk effects, the influence of multiple reflections within acoustic cavities is critical, as they reshape standing-wave patterns, shifting pressure nodes and antinodes, thereby affecting particle trapping stability and control accuracy. In the electrical domain, these reflections influence impedance matching and energy transfer efficiency, altering the overall electro-acoustic array response. Such phenomena have been directly observed in high-frequency sonoreactors, where multiple reflections alter the acoustic field and ultrasonic process efficiency [45]. Similarly, electro-acoustic studies of airborne levitation systems based on ultrasonic transducer arrays confirm that cavity reflections must be carefully considered to accurately predict levitation dynamics and improve performance [46]. These observations highlight the importance of cavity geometry and boundary conditions for optimal levitation stability, control, and system efficiency, complementing the strategies described above to mitigate crosstalk and enhance particle manipulation.

## 3. Influence of Array Technology on Crosstalk

**Figure 2:**
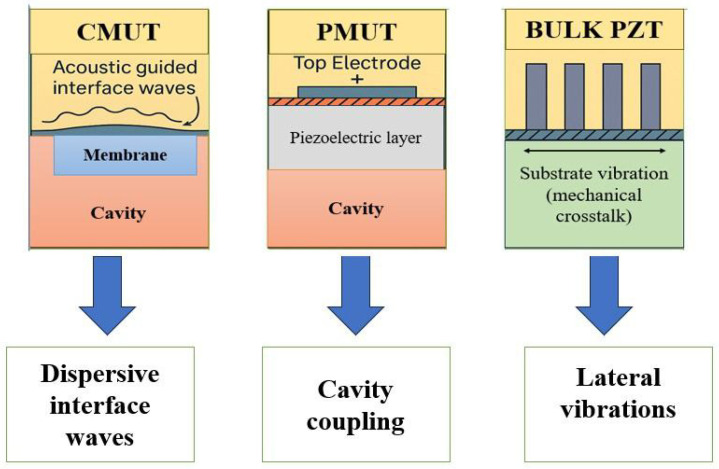
Comparison of dominant crosstalk mechanisms in different transducer technologies: CMUT—dispersive interface waves; PMUT—cavity coupling; Bulk PZT—lateral substrate vibrations.

The extent and nature of crosstalk in ultrasonic transducer arrays are strongly influenced by the specific technology used in their fabrication. Capacitive micromachined ultrasonic transducers (CMUTs), piezoelectric micromachined ultrasonic transducers (PMUTs), and bulk piezoelectric arrays differ significantly in their physical configurations, operating principles, and interaction with surrounding media as illustrated in Figure 2. These differences result in distinct dominant crosstalk mechanisms. This section reviews the technological characteristics of each transducer type and highlights the predominant source of crosstalk in each case.

### 3.1. Capacitive Micromachined Ultrasonic Transducers (CMUT)

CMUTs are MEMS-based devices that consist of flexible membranes suspended above cavities and driven by electrostatic forces. Their wide bandwidth [47] and compatibility with CMOS fabrication make them attractive for high-resolution imaging, particularly in medical diagnostics [48]. However, when operating in fluidic media, CMUTs are particularly prone to acoustic crosstalk due to strong fluid-mediated interactions between adjacent membranes.

A dominant source of this crosstalk is the propagation of dispersive guided modes and Stoneley-type interface waves along the CMUT surface, which significantly disturb neighboring elements [49,50,51,52]. These interactions are exacerbated in conventional operation modes and are further amplified by impedance mismatch at the fluid–solid interface [53]. Additionally, Lamb waves (A0 and S0 modes) may contribute to crosstalk, although their effect is typically secondary [49]. Crosstalk levels vary with the array’s operating mode; for instance, collapse-mode operation significantly attenuates guided modes and lowers inter-element interference. Various mitigation strategies such as silica aerogel layers [54], waveform shaping [14], and membrane decoupling [55] have been proposed to suppress these interactions. Despite their drawbacks, acoustic interactions in CMUTs may also be leveraged beneficially in microfluidic applications [52].

### 3.2. Piezoelectric Micromachined Ultrasonic Transducers (PMUT)

PMUTs are electromechanical resonators composed of piezoelectric thin films and diaphragms that generate acoustic waves upon deformation. They are particularly suited for integration with electronics and feature low acoustic impedance, making them ideal for air-coupled and low-power applications [56].

In PMUT arrays, the dominant form of crosstalk is acoustic, primarily due to air-backed acoustic cavities that facilitate inter-element coupling. Unlike CMUTs, PMUTs exhibit negligible electrical or structural crosstalk; experimental and numerical studies show that these forms of interference are minimal compared to the acoustic contribution [22,57].

Crosstalk arises from pressure wave propagation through acoustically connected cavities, which modify the beam pattern and acoustic output of the array [58]. These interactions are detectable through the emergence of secondary peaks in frequency response, which are significantly reduced by structural isolation techniques or partial decoupling [22]. Interestingly, such coupling effects can also be exploited for remote excitation or frequency filtering applications [30].

The strong impedance mismatch between PMUT membranes and their surrounding media amplifies acoustic transmission, further emphasizing the role of this coupling mechanism in crosstalk [53]. Recently, dual-frequency PMUT designs have been reported, enabling higher sensitivity while minimizing inter-element coupling in biomedical imaging applications [59]. This highlights the ongoing relevance of advanced PMUT architectures for achieving both performance improvement and crosstalk suppression.

### 3.3. Bulk Piezoelectric Transducer Arrays

Bulk PZT arrays are built using monolithic ceramic elements that exhibit high electromechanical coupling and are widely used in applications requiring high acoustic power and efficiency [60]. Compared to CMUTs and PMUTs, they offer robust performance but are more susceptible to mechanical crosstalk due to their solid-state structure.

The most prevalent form of crosstalk in bulk arrays is mechanical vibration transmission across adjacent elements. This occurs due to insufficient mechanical isolation, allowing vibrational modes to propagate through the substrate or shared structures [23,61]. Geometrical parameters—such as element spacing and aspect ratio—and the mechanical properties of the isolation material critically influence crosstalk severity. In addition, electrical boundary conditions, such as open-circuit or short-circuit states of passive elements, significantly affect electromechanical coupling and crosstalk, emphasizing the need for their careful consideration in the design and optimization of high-density PZT arrays [62].

Although mechanical crosstalk dominates in bulk PZT arrays, capacitive coupling can also contribute to electrical crosstalk, typically as a secondary effect arising from parasitic capacitances in packaging or interconnects [63]. Techniques such as voltage feedback loops and zero-potential grounding circuits have been proposed to suppress this form of interference [64]. Despite the dominance of mechanical crosstalk, acoustic crosstalk may also arise under 231 specific conditions, especially in miniaturized versions of PZT transducers, such as PMUTs, 232 where air cavities exist between elements [22]. As shown in Table 1, various ultrasonic transducer technologies exhibit distinct crosstalk mechanisms and corresponding mitigation strategies.

### 3.4. Emerging Technologies

Beyond conventional PMUT, CMUT, and bulk piezoelectric technologies, several emerging ultrasonic transducer designs have been proposed to mitigate crosstalk, which remains a critical limitation in array-based systems. Silica aerogel coatings applied to CMUT arrays have demonstrated substantial suppression of Scholte wave-induced crosstalk through impedance mismatch at the fluid–device interface, leading to an average 22.1% improvement in −6 dB fractional bandwidth performance [54]. Hybrid micromachined ultrasonic transducers (HMUTs), integrating both piezoelectric and electrostatic actuation mechanisms, have shown over a ninefold improvement in transmit sensitivity and significantly reduced inter-element interference [65]. Dual-frequency PMUTs, leveraging a dual-electrode configuration on a single diaphragm, exhibit nearly negligible crosstalk in pulse-echo experiments, making them promising for high-resolution biomedical imaging [21]. Phononic crystals (PNCs), which introduce frequency bandgaps to inhibit wave propagation, have achieved up to 40 dB crosstalk attenuation in ultrasonic flowmeters [66]. Additionally, resonant cavity-based PMUT arrays and sol–gel composite spray techniques have demonstrated enhanced output sensitivity and suppressed adjacent-element coupling down to −41.5 dB, respectively [67,68]. These innovative approaches highlight the importance of tailored design strategies for effective crosstalk mitigation, balancing performance enhancement with fabrication complexity and application-specific constraints. More recently, Oh et al. (2025) proposed a patch-type CMUT for implantable devices, demonstrating that effective crosstalk suppression remains critical not only for imaging performance but also for reliable ultrasonic power and data transfer in biomedical applications [69].

### 3.5. Ferroelectret-Based Transducers

Ferroelectret ultrasonic transducers, based on charged polymer films with internal cavities acting as macroscopic dipoles, are emerging as a promising technology for both medical imaging and air-coupled applications. They combine lightweight, flexible, and cost-effective designs with strong piezoelectric-like behavior, low acoustic impedance, wideband response, and high sensitivity. Studies have demonstrated broad frequency operation (0.3–2.5 MHz) with relative bandwidths exceeding 100% and potential for dual-frequency operation, enabling advanced imaging techniques such as harmonic imaging and image-guided therapy [59,70]. Flexible polymer implementations (e.g., PVDF-TrFE) allow large-aperture and real-time imaging [71], while airborne phased arrays have shown promise for efficient fabrication and reduced system complexity [72,73]. For example, for airborne ultrasonic phased arrays using ferroelectrets, a new fabrication approach [74] demonstrated their potential for efficient array fabrication and reduced complexity. Despite these advantages, challenges remain, including limited structural durability, impedance-matching trade-offs affecting SNR, and non-uniform frequency responses in phased arrays. Strategies such as additional biasing for sensitivity enhancement [75], 3D printing with biocompatible polymers [76], innovative assembly techniques, and FPGA-based signal processing [73] have been proposed to overcome these limitations. Overall, ferroelectret transducers offer a unique combination of material properties and design flexibility, making them a valuable complement to CMUTs, PMUTs, and bulk PZT arrays in next-generation ultrasonic applications.

## 4. Characterization Techniques

Crosstalk represents a major challenge in the design of ultrasonic transducer arrays, particularly in high-resolution applications such as medical imaging. It manifests as undesired coupling of energy between adjacent elements, disrupting beam directivity, sensitivity, and signal fidelity. Several approaches have been developed to characterize and model this phenomenon, each offering distinct advantages depending on the transducer technology (CMUT, PMUT, bulk piezoelectric, etc.) and the required level of modeling detail. These approaches are commonly grouped into experimental methods, numerical modeling, hybrid analytical models, and equivalent circuit methods.

### 4.1. Experimental Approaches

Direct measurement techniques enable empirical characterization of crosstalk by analyzing captured signals and frequency responses in neighboring elements. These methods are also used to validate numerical models. The analysis of signal decay and propagation velocities of parasitic waves is commonly used in this context [13,26]. Another important technique for characterizing mechanical crosstalk is laser interferometry. This non-contact optical method makes it possible to measure vibration profiles of the transducer surface with nanometric precision. By mapping these vibrations, it becomes easier to identify unintended resonances and coupling between neighboring elements. Caronti et al. (2003) successfully applied laser interferometry to CMUT arrays, showing how guided wave propagation could be quantified and compared with FEM simulations [51]. This approach provides a valuable complement to impedance and direct signal measurements, especially when investigating the mechanical origin of crosstalk. Moreover, laser interferometry enables the detection of abnormally responsive or non-responsive elements, supporting the optimization of transducer design and performance [77,78,79]. However, despite its high precision, this technique remains sensitive to environmental disturbances, technically complex, and costly and is therefore of limited applicability in clinical environments where stability cannot be ensured [78,80]. To address these challenges, advanced approaches such as pulsed restored-coherence interferometry, improved materials, and innovative signal processing techniques have been proposed, including FMCW interferometry for error reduction [81], heterodyne systems for broadband acoustic pressure measurement [78], homodyne self-calibration for sub-nanometer resolution [82], two- and three-dimensional displacement detection [81,83], and optical grating diffraction systems for ultra-high precision [84]. Together, these advances highlight laser interferometry as a promising but technically demanding tool for improving the accuracy and reliability of mechanical crosstalk characterization in medical ultrasound imaging [25,85]. Electrical impedance characterization also provides valuable insights into parasitic electromechanical effects contributing to crosstalk [86].

**Figure 3:**
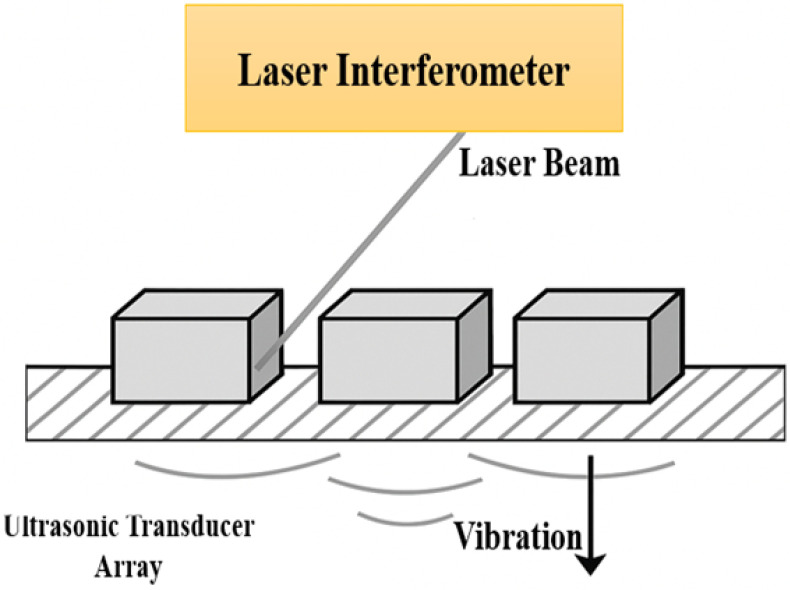
Schematic illustration of laser Doppler interferometry applied to ultrasonic transducer arrays.

Figure 3 shows how the laser beam is directed onto the surface of individual array elements, and the reflected signal is analyzed to capture nanometric vibrations. This non-contact technique enables precise characterization of mechanical crosstalk by mapping vibration profiles and detecting unintended coupling between neighboring elements. Such methods have been successfully applied, for instance, in CMUT arrays to quantify guided wave propagation and validate FEM predictions [51].

### 4.2. Numerical Methods (FEM, BEM, HPC)

The Finite Element Method (FEM) is the most widely used technique to simulate complex interactions between transducer elements. It enables the assessment of mechanical and electroacoustic crosstalk, the identification of parasitic resonance modes, and exploration of the effects of materials and geometries on array performance [4,25].

Hybrid FEM/analytical methods have been used to model infinite periodic arrays [87], while cloud-based High-Performance Computing (HPC) combined with FEA enables the simulation of large-scale PMUT arrays, particularly to study the effects of frequency and pitch spacing on crosstalk [88]. The Fast Multipole Algorithm (FMA) improves the computational efficiency of boundary element methods for membrane-based MUT structures [89].

### 4.3. Simplified Analytical Models and Equivalent Circuits

Equivalent circuit methods allow rapid and intuitive modeling of crosstalk phenomena, particularly during early-stage design and optimization phases [90,91]. These models effectively capture mutual acoustic interactions and impedance characteristics while reducing computational cost and degrees of freedom [92,93,94]. They are especially suitable for regular array structures such as CMUT or bulk piezoelectric arrays and can be integrated with other numerical techniques.

### 4.4. Directivity-Based Models

The analysis of beam directivity patterns helps identify and quantify the impact of crosstalk on array radiation behavior. Distortions in the radiated field, particularly at large steering angles, are strong indicators of undesired coupling. These models also support the development of mitigation strategies such as injecting compensating voltages or optimizing structural design [13,18,27].

### 4.5. Quantitative Metrics for Crosstalk Evaluation

Several quantitative metrics have been employed in the literature to assess the severity and characteristics of crosstalk in ultrasonic transducer arrays. These indicators are critical for benchmarking the effectiveness of both passive and active mitigation techniques. The most commonly reported metrics include the following:

- Decay Rate of Parasitic Signals: used to quantify how rapidly unintended signals diminish over distance or time, offering insight into spatial or temporal leakage characteristics [13].
- Normalized Complex Impedance: the inter-element impedance, normalized with respect to reference values, provides a sensitive measure of electrical coupling and has been widely applied in both CMUT and PMUT technologies [86,95].
- Directivity Pattern Distortion: evaluated by comparing the ideal and perturbed beam patterns, this metric helps identify spatial deviations caused by mutual interactions among elements [27].
- Parasitic Guided Wave Propagation: the presence and propagation of guided parasitic waves within the substrate or matching layers is often analyzed through simulation or measurement-based methods [49,96].
- Amplitude Variation Between Neighboring Elements: a practical and intuitive indicator, especially in array configurations, where unintended excitation of adjacent elements can be directly measured.
- Parasitic Resonance Levels Across Frequency: the presence of resonances unrelated to the desired operating mode, as a function of frequency, serves as a strong indicator of structural or acoustic crosstalk [97].

These metrics collectively provide a comprehensive understanding of crosstalk mechanisms and support the development of robust diagnostic and mitigation strategies.

Table 2 provides a concise overview of key crosstalk characterization techniques across ultrasonic transducer technologies such as CMUT, PMUT, and bulk piezo. Methods range from direct experimental measurements to advanced numerical simulations and equivalent circuit models, reflecting the multifaceted nature of crosstalk involving coupled mechanical, electrical, and acoustic effects. The use of high-performance computing and accelerated algorithms underscores recent advances in efficiently modeling large complex arrays. Collectively, these approaches form a comprehensive toolkit for precise crosstalk diagnosis and targeted mitigation strategy development.

Section 5 details these strategies, highlighting their effectiveness, limitations, and suitability for different array technologies and application scenarios, as summarized in Table 3 and Table 4. This integrated approach ensures a coherent connection between characterization results and practical crosstalk reduction methods.

## 5. Crosstalk Reduction Strategies

### 5.1. Passive Crosstalk Reduction Techniques

**Figure 4:**
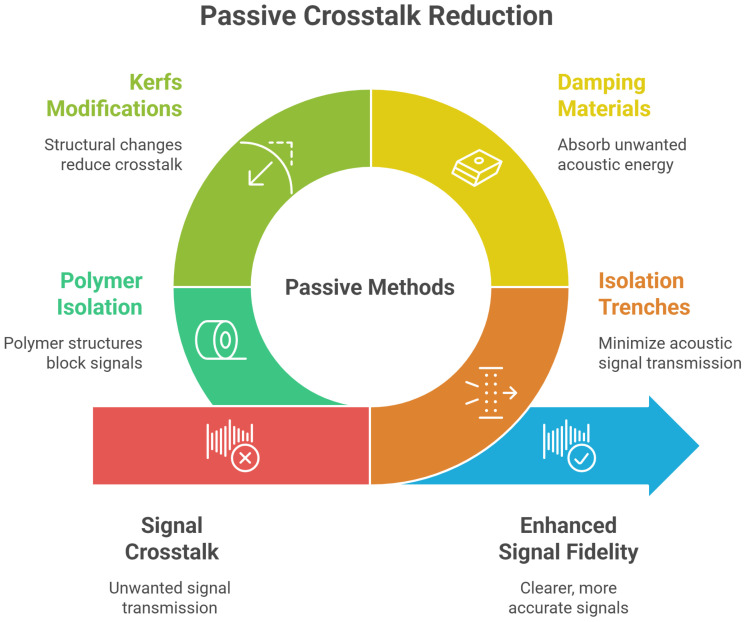
Passive crosstalk reduction methods.

Passive methods refer to structural and material-based strategies that mitigate crosstalk in ultrasonic transducers without relying on active electronic components. These techniques are often favored for their simplicity, reliability, and ease of integration into existing transducer designs. They aim to minimize the transmission of unwanted acoustic, mechanical, or electrical signals between adjacent elements, thereby enhancing the signal fidelity, imaging quality, and overall device performance. Passive crosstalk reduction techniques can be broadly categorized into four classes: (a) isolation trenches, (b) damping materials, (c) kerfs and structural modifications, and (d) polymer-based isolation structures (Figure 4).

#### 5.1.1. Isolation Trenches

Isolation trenches are one of the most widely adopted methods, particularly effective in CMUT and PMUT arrays. They work by physically separating transducer elements, thereby disrupting the propagation of inter-element surface and guided waves. Studies have demonstrated that such trenches effectively block Stoneley and Lamb waves, which are known contributors to acoustic crosstalk in ultrasonic devices [15,96]. In CMUT architectures, the implementation of trench-isolated electrodes has achieved reductions in electrical crosstalk down to −53 dB at 5 MHz [106]. For PMUTs, trench integration led to a 76% increase in output pressure while preserving the device’s resonance frequency, a result attributed to the reduction in tensile stress at membrane edges [107]. These results confirm the effectiveness of trench-based approaches in simultaneously enhancing the acoustic and electrical performance while maintaining structural integrity and ease of fabrication. However, their implementation introduces several practical challenges. Fabrication requires precise deep reactive-ion etching (DRIE) and advanced lithography, significantly increasing the complexity and cost [108,109]. The performance is highly sensitive to the trench depth, width, and material choice, affecting electrical, thermal, and mechanical properties. Additionally, trenches can induce mechanical stress concentrations in the substrate, potentially reducing device durability, and may slightly attenuate signal strength or impact high-frequency resolution [110,111]. These factors necessitate careful design optimization, and in practice, trench-based isolation is often combined with complementary passive or active techniques to achieve optimal crosstalk mitigation while balancing fabrication feasibility, performance, and reliability.

#### 5.1.2. Kerfs and Structural Modifications

Kerfs and structural modifications offer a more straightforward yet highly effective means of reducing both acoustic and electrical crosstalk. By introducing mechanical discontinuities through cuts between adjacent piezoelectric elements, these modifications disrupt mechanical coupling. Deeper kerfs that penetrate into the interfacial layer have been shown to enhance both acoustic and dielectric isolation [112]. Finite element simulations and experimental validations have confirmed that kerf geometry and material composition significantly influence the isolation performance [15,96]. Nonetheless, kerfs can also introduce stress concentrations and cause shifts in the resonance frequency of the transducer, potentially impairing its operating efficiency [113,114]. Additionally, such modifications may degrade the dielectric properties of the transducer material, leading to increased electrical losses and signal distortion, particularly in high-frequency or low-noise applications [24]. While straightforward to implement, the mechanical and electrical trade-offs of kerf-based designs necessitate careful design optimization.

##### Isolation Trenches vs. Kerfs

**Figure 5:**
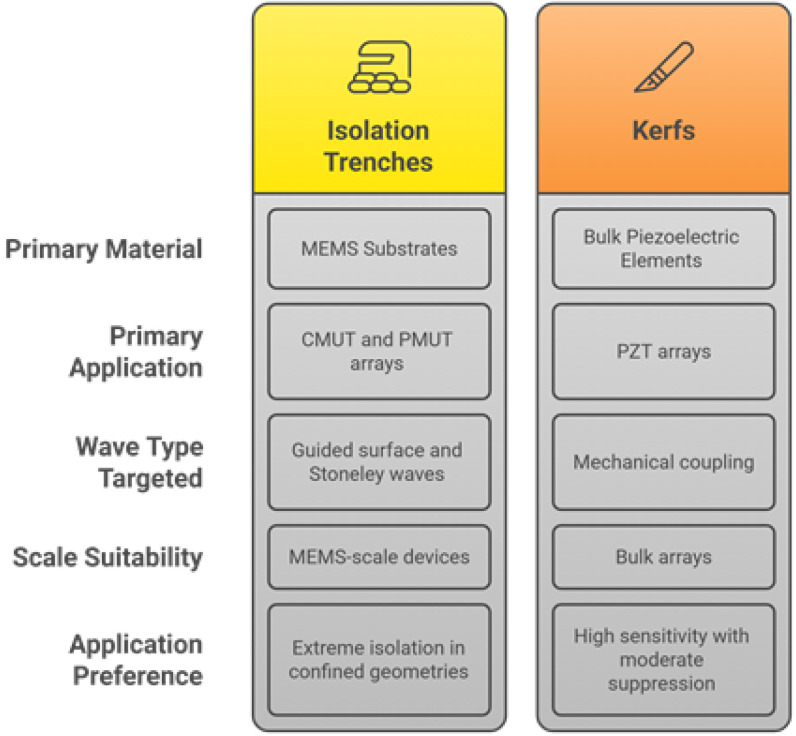
Comparative performance of isolation trenches and kerfs in reducing acoustic and mechanical crosstalk in ultrasonic transducer arrays.

Although isolation trenches and kerfs are sometimes mentioned interchangeably, they differ fundamentally in implementation and application. Isolation trenches are micro-fabricated separations commonly used in CMUT and PMUT arrays, designed to block guided surface and Stoneley waves within MEMS substrates. They are particularly effective in high-precision applications, such as intraluminal medical imaging, by minimizing acoustic coupling through shared substrates [57,115]. Kerfs, on the other hand, are mechanical cuts introduced between bulk piezoelectric elements, primarily in PZT arrays, to minimize mechanical coupling across the ceramic substrate [85]. While both approaches aim at reducing crosstalk, trenches are more suited to MEMS-scale devices where precision etching is feasible, whereas kerfs remain the standard solution for bulk arrays where mechanical robustness is critical; trenches are generally preferred for applications requiring extreme isolation in confined geometries, whereas kerfs are suitable when maintaining high sensitivity alongside moderate crosstalk suppression. Alternative approaches, such as aerogel layers or single-crystal configurations, can further enhance crosstalk reduction, achieving improvements of 31.5% and 6–8.8 dB, respectively [54,116] (Figure 5).

#### 5.1.3. Damping Materials

The use of damping materials represents another passive approach that addresses crosstalk by targeting energy dissipation mechanisms. These materials absorb and convert acoustic energy, often into heat, thereby reducing spurious signal propagation within the transducer housing. In ultrasonic gas flowmeters, matrix composites containing suspended particles have been shown to reduce both noise and crosstalk, enabling more accurate signal detection in gaseous environments [117]. Materials designed to match the acoustic impedance of the surrounding structure further help minimize reflection and energy buildup, as shown in composites incorporating tungsten additives [118]. Some damping layers incorporate conductive particles within a piezoelectric matrix to convert acoustic energy into thermal energy, which has proven effective in high-precision environments [119]. This approach is commonly used in imaging devices, where fluid damping layers applied to the transducer interior have improved image clarity by suppressing internal reverberations [120]. However, the risk of excessive damping in ultrasonic and MEMS transducers can degrade both the sensitivity and resolution, reducing the dynamic response and signal detection capability [121,122,123,124,125]. It may also alter the mechanical and electrical characteristics, introducing impedance in CMUTs and suppressing essential electromechanical resonances in piezoelectric actuators [126,127]. Careful optimization is therefore required to balance the noise suppression and resonance control with the performance, particularly in high-frequency or MRI-compatible applications [128].

#### 5.1.4. Polymer-Based Isolation Structures

**Figure 6:**
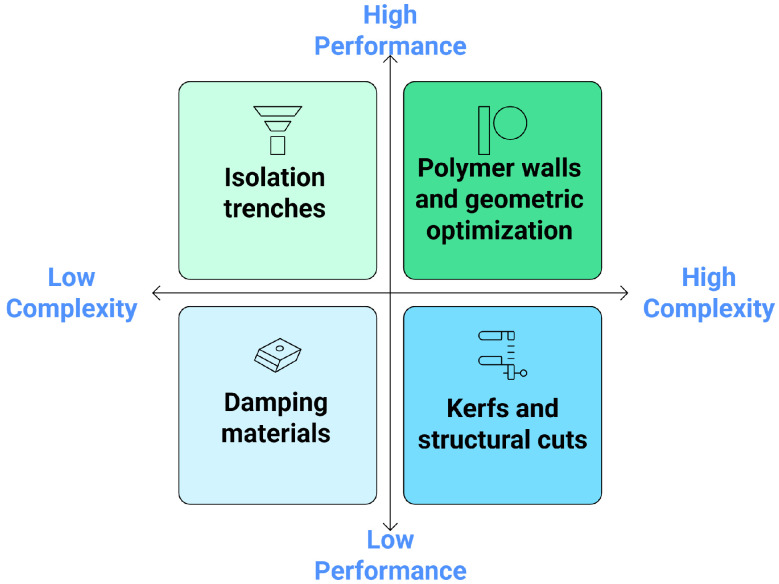
Trade-offs in ultrasonic transducer isolation techniques.

A more advanced approach involves the strategic use of polymer-based isolation structures to modify acoustic wave propagation paths. These include polymer walls placed between elements, mesas, ring-shaped supports, and pseudo-random pillar geometries in piezocomposite arrays. The geometric parameters of these structures—particularly the width, depth, and shape—have a more pronounced effect on crosstalk reduction than the intrinsic material properties [15,96]. Convex and concave geometries, for example, can reshape wave propagation by either dispersing or focusing reflected signals, with convex structures helping to reduce concentrated crosstalk through spatial redistribution [129]. In high-frequency applications, pseudo-random geometries disrupt coherent wave patterns, yielding lower crosstalk levels than traditional square or triangular layouts [130]. In PMUTs and CMUTs, the inclusion of polymer-filled trenches and membrane separators has also been effective in suppressing dispersive guided modes, further improving inter-element isolation [55]. These geometric approaches, while powerful, often demand sophisticated fabrication techniques and precise lithographic control, particularly for dense array configurations. The comparison of passive isolation methods and their associated trade-offs is summarized in Figure 6.

Recent research has extended passive strategies through innovative material and structural solutions aimed at mitigating crosstalk in ultrasonic transducers. For CMUT arrays, the application of nanoporous silica aerogel coatings enriched with diisocyanate has demonstrated a notable 31.5% improvement in crosstalk suppression at 7.5 MHz by effectively attenuating Scholte waves at the CMUT–fluid interface [54]. Piezoelectric composites, such as PZT/epoxy, have also been optimized to reduce inter-element interference, with fine-tuning of kerf dimensions enabling crosstalk reduction up to 24.1 dB [17]. Sol–gel spray techniques have facilitated the fabrication of thin, flexible, and biocompatible transducers, achieving exceptionally low crosstalk levels, as low as 45.8 dB between adjacent elements [68].

#### 5.1.5. Recent Material and Structural Innovations

In PMUT arrays, structural modifications including rectangular grooves and deliberate misalignment of elements have proven effective in lowering vibrational coupling from 44.5% to 14.8% [21,131], while the integration of dielectric insulating layers such as polyimide or high-k materials like hafnium oxide (HfO_2_) not only enhances electromechanical coupling but also contributes to crosstalk reduction [132,133]. Designs incorporating resonant cavities have further improved performance by redirecting parasitic acoustic waves away from neighboring elements, leading to a remarkable 259% increase in output sensitivity compared to conventional arrays [67]. Additionally, phononic crystals (PNCs) have attracted attention for their ability to create frequency bandgaps that inhibit the propagation of undesired acoustic waves, achieving up to 40 dB reduction in ultrasonic flow meter applications while also filtering nonlinear harmonic waves and isolating acoustic energy in sensor arrays [66,134,135]. Despite their advantages, the practical implementation of PNCs is often limited by fabrication complexity and associated costs.

#### 5.1.6. Comparative Analysis

A comparative analysis of these techniques highlights their respective strengths and limitations (Table 3). Isolation trenches and polymer walls are most effective in micromachined transducers like CMUTs and PMUTs, while kerfs are well suited for bulk PZT arrays. Damping materials offer broadband suppression, particularly in systems where resonance ringing and mechanical echoes are problematic. However, excessive damping may degrade signal fidelity. Polymer geometry manipulation provides high design flexibility and spatial control but requires high-precision fabrication and simulation-based optimization.

**Figure 7:**
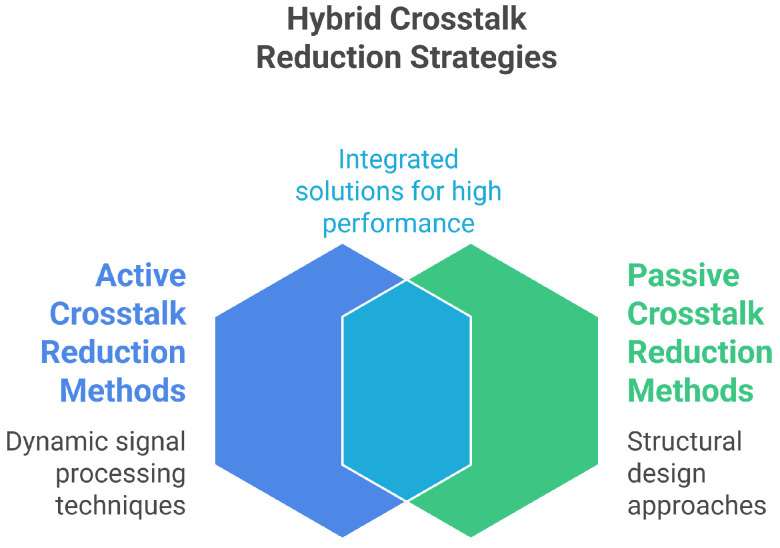
Hybrid crosstalk reduction methods.

While passive approaches alone can significantly reduce crosstalk, hybrid strategies that combine them with active waveform shaping [14] or temporal isolation techniques [136] as illustrated in Figure 7 offer greater flexibility and dynamic control. These integrated solutions may enhance performance further without significant manufacturing trade-offs.

### 5.2. Active Crosstalk Reduction Methods

**Figure 8:**
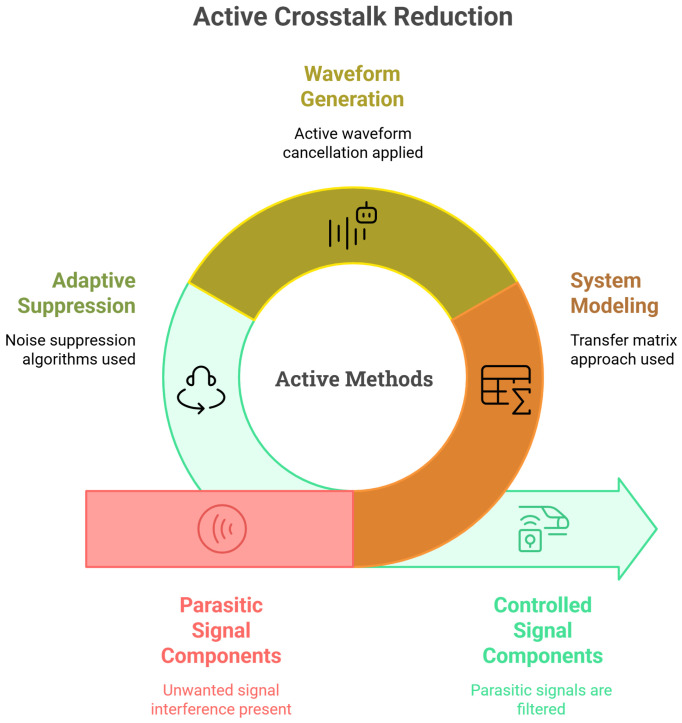
Active crosstalk reduction methods.

Active methods aim to dynamically control the transmitted and received signals in order to compensate for, cancel, or filter out the parasitic signal components, either as a complement or an alternative to structural passive modifications. These approaches generally rely on adaptive signal processing techniques, optimized waveform generation, corrective voltage application, or matrix-based system modeling. For clarity, we classify them into five main categories: (a) transfer matrix-based modeling, (b) active waveform generation and cancellation techniques, (c) corrective voltage-based reduction, (d) active impedance matching or latching schemes, and (e) adaptive crosstalk cancellation algorithms. The comparison of active crosstalk reduction techniques is presented in Figure 8.

#### 5.2.1. Transfer Matrix-Based Modeling

The transfer matrix approach models the transducer array using a transfer function matrix that captures the effect of one excited element on all others. By inverting this matrix, one can derive the optimal excitation waveforms for each element to minimize the apparent crosstalk. Zhou et al. [98] demonstrated the effectiveness of this method in reducing sidelobes and focusing angular responses, achieving up to 9 dB reduction in CMUT arrays simulated via finite element modeling. This method was also successfully validated on physical prototypes, showing significant improvements in beam directivity [98]. Its key advantage lies in its ability to explicitly model parasitic interactions across the array. However, it requires precise system calibration and entails a high computational cost, which can be a limiting factor in real-time implementations. While highly effective in calibrated and stable environments, this approach becomes difficult to generalize to irregular geometries or dynamically varying systems, as noted by Lambert et al. [137].

#### 5.2.2. Active Waveform Generation and Cancellation Techniques

**Figure 9:**
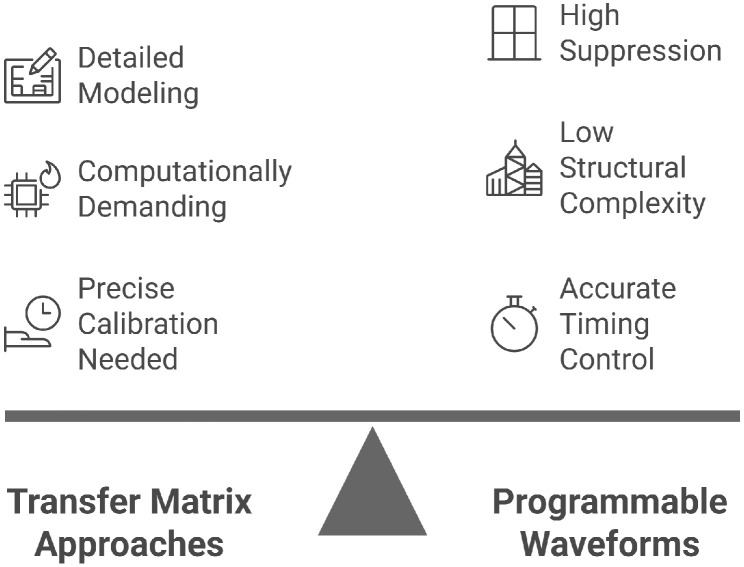
Comparing crosstalk active reduction techniques: transfer matrix approaches and programmable waveforms.

These methods aim to dynamically tailor the input signals to each element to actively suppress crosstalk components. Programmable waveform synthesis allows individual signal shaping per element to compensate for inter-element coupling. Using this strategy, Zhou and Hossack [14] reported a reduction of up to 25 dB in CMUT arrays under low-voltage AC excitation. Another technique, harmonic iterative cancellation, refines crosstalk suppression by iteratively correcting harmonic distortions in the transmitted signal, yielding up to 25.5 dB of suppression [14]. This method, while highly effective in reducing crosstalk, often requires advanced electronic control systems and is sensitive to waveform synchronization precision and signal stability. The practical implementation of these methods therefore depends heavily on the quality of hardware and timing control, which may limit their robustness in certain application contexts. Figure 9 compares active crosstalk reduction techniques, including transfer matrix approaches and programmable waveforms.

#### 5.2.3. Active Reduction Using Corrective Voltages

Another active strategy is based on applying corrective voltages to neighboring elements in order to nullify parasitic vibrations caused by inter-element coupling. This concept was investigated numerically by Cugnet et al. [99], who developed a finite element-based numerical technique demonstrating the theoretical feasibility of canceling undesired displacements in acoustical arrays. Building on these approaches, Bybi et al. [100] conducted a feasibility study showing that crosstalk can be diminished by superimposing compensating voltages on neighboring elements, calculated from the superposition principle of displacements. Piezoelectric array prototypes (PZT27) were fabricated and tested, and an experimental reduction of about 6–10 dB was observed, even on asymmetrical devices with fabrication defects. This method was later extended to the transient regime by Bybi et al. [101], where a two-dimensional finite element model (ATILA) of a 17-element array was analyzed. Corrective signals were computed using FFT/inverse FFT processing, leading to reductions of up to 30 dB depending on the element position, along with a significant improvement of the directivity pattern, with the main lobe re-centered on the axis.

Overall, corrective voltage techniques present significant advantages, as they enable the active suppression of both electrical and mechanical crosstalk without requiring physical modifications of the array geometry. Their adaptability to fabrication tolerances and varying operational conditions, combined with the possibility of implementation using relatively simple sensing circuitry based on impedance or motional current measurements, makes them particularly attractive compared to displacement-based methods. Moreover, these approaches contribute to an improvement in beam directivity and overall image quality, which is especially valuable for applications in medical imaging and non-destructive testing. Despite these strengths, several limitations persist. Most demonstrations have been performed on small arrays (5–17 elements) [100,101], which raises concerns regarding scalability to clinical systems incorporating hundreds of elements. In addition, the reliance on precise synchronization and calibration can increase the hardware complexity and may compromise stability under real-time operating conditions. While reported suppression levels typically reach 20–30 dB, these improvements may still fall short of the requirements for high-resolution imaging, where residual crosstalk can significantly affect sensitivity and contrast [138]. Future work should therefore prioritize the development of scalable implementations for high-element-count arrays, the integration of corrective voltage methods with advanced beamforming algorithms, and thorough validation under realistic clinical conditions.

#### 5.2.4. Active Impedance Matching/Latching Techniques

Another class of active methods focuses on electronic impedance adaptation to further reduce crosstalk. In this domain, impedance matching techniques have emerged as effective strategies by optimizing acoustic energy transfer and minimizing reflections within the transducer array. Techniques employing negative capacitance circuits have been demonstrated to improve bandwidth and reduce acoustic reflections significantly, leading to lower crosstalk levels in CMUT arrays with configurations of up to 16 elements [138]. Dynamic impedance matching systems, utilizing variable component L-type circuits, offer real-time optimization of electroacoustic conversion efficiency, resulting in reduced reflected power and enhanced acoustic pressure levels [139]. These methods underscore the critical role of electronic adaptation in complementing material and structural innovations to achieve comprehensive crosstalk suppression.

#### 5.2.5. Adaptive Crosstalk Cancellation

This signal processing-based strategy aims to filter out the parasitic signal components in real time using adaptive filtering techniques, particularly those based on normalized least-mean-squares (NLMS) algorithms. Nguyen et al. [140] introduced an adaptive suppression technique in the slowness-time domain, allowing efficient separation of useful signals from interfering components. These techniques are robust to variations in propagation conditions and can adapt in real time to environmental changes, making them well suited for dynamic imaging scenarios.

**Figure 10:**
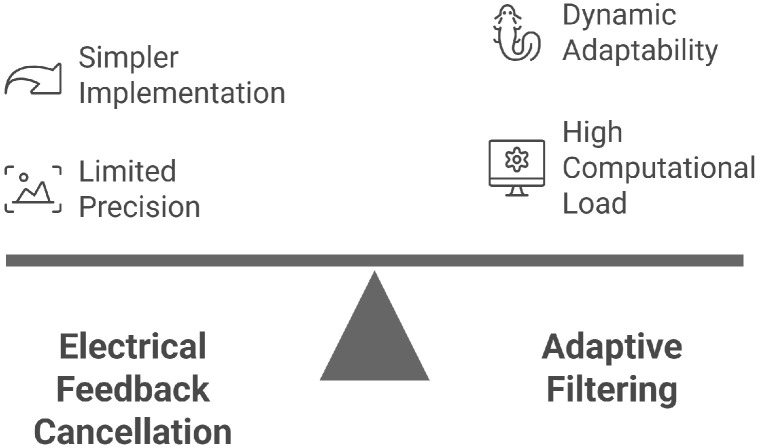
Comparing crosstalk active reduction techniques: electrical feedback control and adaptive filtering.

However, their implementation requires substantial computational resources and relies on an accurate reference model of the desired signal. Advanced algorithmic solutions—also classified as active methods—based on adaptive filtering, notably the Least Mean Squares (LMS) algorithm, have shown great promise in attenuating crosstalk through real-time signal processing. LMS-based techniques dynamically adjust filter coefficients to minimize the error between desired and actual outputs, providing an adaptive and robust framework for crosstalk cancellation. Lu et al. [102] demonstrated that LMS algorithms could eliminate up to 88.9% of crosstalk noise in narrowband signals and as much as 98.53% in broadband applications when combined with band-split filtering, reducing residual noise to as low as 1.47%. Moreover, the extension of these techniques to matrix representations of transfer functions enables precise excitation control in multi-element arrays, facilitating further reductions in interelement interference [141]. Figure 10 compares active crosstalk reduction techniques, including electrical feedback control and adaptive filtering.

Despite challenges such as convergence speed limitations and sensitivity to analog-to-digital conversion accuracy, LMS-based methods remain highly valuable for dynamic real-time crosstalk mitigation, particularly in complex multi-channel systems [142,143].

Active methods for crosstalk reduction offer greater flexibility and performance compared to passive techniques, especially in modern CMUT and PMUT arrays (Table 4). However, their effectiveness is often contingent upon external factors such as system stability, synchronization precision, and calibration accuracy. In practice, hybrid strategies that combine active signal processing with passive structural design often provide the best trade-off in demanding scenarios such as high-resolution medical imaging. The optimal method should be selected based on the desired compromise between system complexity, cost, performance, and adaptability.

**Figure 11:**
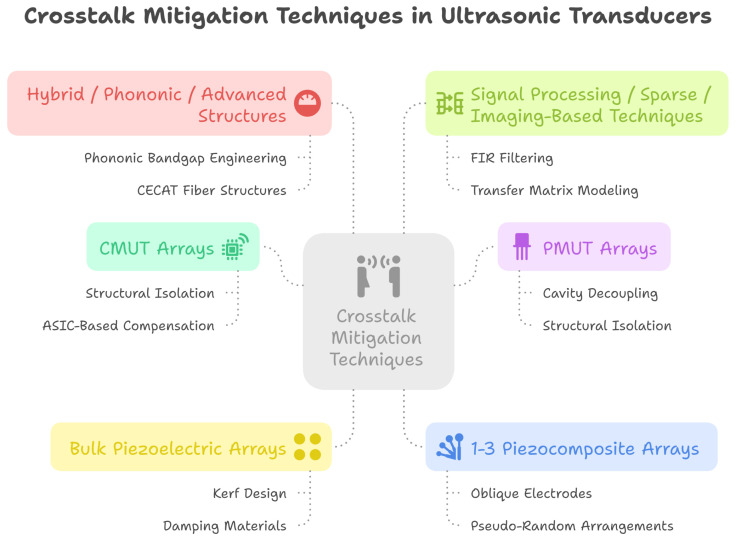
Crosstalk reduction techniques in ultrasonic transducers.

In summary (Figure 11), recent advancements in crosstalk reduction combine passive and active strategies. Passive methods—including material innovations, structural modifications, and phononic crystals—have shown significant improvements: CMUT arrays benefit from aerogel coatings, PZT/epoxy composites, and thin sol–gel transducers, while PMUT arrays achieve superior performance through structural grooves, deliberate misalignment, dielectric insulation, and resonant cavities. Phononic crystals further enhance suppression by creating frequency bandgaps and isolating acoustic energy. Active approaches, such as impedance matching using negative capacitance or dynamic L-type circuits and adaptive LMS filtering, optimize energy transfer, minimize reflections, and cancel parasitic signals in real time. Electrical and geometrical suppression strategies have also been demonstrated in row–column actuator arrays. Together, these complementary methods allow tailored solutions for different transducer types, balancing crosstalk reduction, sensitivity, and practical considerations. Integrating passive and active techniques while accounting for fabrication complexity and application-specific trade-offs remains essential for advancing ultrasonic transducer performance and improving imaging and sensing capabilities. A comprehensive summary of the reported crosstalk reduction methods is provided in Table 5.

## 6. Conclusions

This review has systematically addressed the phenomenon of crosstalk in ultrasonic transducer arrays for medical imaging, from its physical origins to its detrimental effects on image clarity and diagnostic reliability. A particular emphasis was placed on characterization techniques—both experimental and numerical—providing the necessary foundation for evaluating and comparing mitigation strategies.

One of the central outcomes of this work is the clear demonstration that the effectiveness of a crosstalk reduction method is strongly dependent on the underlying transducer technology. Capacitive micromachined ultrasonic transducers (CMUTs), piezoelectric micromachined ultrasonic transducers (PMUTs), and bulk PZT-based transducers each exhibit distinct crosstalk mechanisms—acoustic interface waves, cavity coupling, or substrate vibrations—that require tailored solutions. As a result, passive approaches such as aerogel coatings or phononic crystal layers show excellent compatibility with CMUTs, while structural decoupling and dielectric layering are more effective for PMUTs. Conversely, kerf optimization and damping strategies are most suitable for conventional bulk arrays.

Active reduction techniques, including adaptive filtering and impedance matching, also show technology-dependent performance. CMUT arrays, with their capacitive nature, particularly benefit from electronic cancellation and waveform engineering, whereas bulk piezo arrays profit more from mechanical isolation and hybrid circuit models.

This review thus supports the conclusion that the selection of an appropriate crosstalk mitigation strategy must be closely aligned with the specific physical and operational characteristics of the transducer technology used. This insight is especially relevant in medical imaging, where requirements for resolution, sensitivity, and probe compactness vary significantly between applications (e.g., intravascular, abdominal, or musculoskeletal imaging).

By mapping current solutions to transducer types, and identifying open challenges such as the trade-off between isolation and sensitivity—particularly in filler materials—this work provides a structured framework to guide the design of next-generation imaging arrays. Future research should prioritize technology-specific hybrid solutions that combine structural, material, and electronic innovations to meet the increasingly demanding performance requirements of medical ultrasound.

## Figures and Tables

**Table 1:** Comparison of crosstalk mechanisms and mitigation strategies across ultrasonic transducer technologies.

Technology	Key Characteristics	Crosstalk Type	Main Causes	Mitigation Techniques
CMUT	MEMS-based, wide bandwidth, fluid-coupled	Acoustic	Dispersive guided modes, interface waves	Aerogel layers, waveform shaping, structural decoupling
PMUT	CMOS-compatible, low impedance, air-backed	Acoustic	Coupling via air cavities, pressure waves	Partial/full decoupling, cavity redesign
Bulk PZT	High power, robust ceramics, solid state	Mechanical	Substrate vibration, poor isolation	Mechanical damping, element spacing, isolation materials

**Table 2:** Summary of crosstalk characterization methods and associated technologies.

Method	Main Description	Target Technologies	References
Direct Measurement	Time/frequency domain analysis (e.g., decay rate, ToF, impulse response)	CMUT, PMUT, Bulk	Ramalli et al., 2019 [13]; Opielinski et al., 2014 [26]
Impedance Characterization	Detection of parasitic electromechanical effects	CMUT, MEMS	Savoia et al., 2017 [86]; Wang et al., 2016 [95]
Finite Element Method (FEM)	Coupled mechanical–electrical simulations for resonance and vibration modes	Bulk, CMUT, PMUT	Celmer and Opielinski, 2016 [25]; Reskal et al., 2024 [4]
HPC + FEA	Large-scale simulation of MEMS arrays using high-performance computing	PMUT	Pirouz et al., 2019 [88]
Fast Multipole Algorithm (FMA)	Accelerated boundary element modeling for large systems	CMUT	Shieh et al., 2016 [89]
Hybrid FEM/Analytical Models	Efficient modeling of periodic/infinite structures	Bulk	Ventura et al., 1992 [87]
Equivalent Circuit Models	Compact models with electrical analogs of mechanical behavior	CMUT, Bulk, PMUT	Pyo and Roh, 2017 [92]; Li et al., 2024 [94]
Directivity Pattern Analysis	Angular response mapping to detect field distortion	All	Bybi et al., 2013 [27]; Mo et al., 1990 [18]

**Table 3:** Comparison of passive crosstalk reduction techniques.

Method	Targeted Crosstalk Type	Best Suited For	Advantages	Limitations
Isolation Trenches	Acoustic and Electrical	CMUTs, PMUTs	Strong isolation; compatible with MEMS integration	Performance sensitive to trench depth and material selection
Damping Materials	Acoustic	Imaging, flow metering	Broad-band attenuation; helps control resonances	May cause over-damping, reducing device sensitivity
Kerfs and Structural Cuts	Acoustic and Electrical	Bulk PZT Arrays	Simple to fabricate; effective crosstalk reduction	Alters resonance characteristics and may introduce mechanical stress
Polymer Walls and Geometry	Acoustic	High-frequency dense arrays	Precise design control; enables flexible and compact layouts	Requires complex modeling and careful layout optimization

**Table 4:** Comparison of active crosstalk reduction methods.

Method/Study	Crosstalk Reduction Efficiency	Implementation Complexity	Impact on Image Quality/Advantages	References
Transfer matrix approach	9–25 dB	Moderate; requires transfer function matrix and waveform optimization	Reduced sidelobes and improved angular response	Zhou et al. [98]
Programmable waveforms	20–25.5 dB	High; involves programmable waveform transmit circuits	Significant crosstalk suppression maintaining linear operation	Zhou et al. [14]
Corrective Voltages—FEM (theoretical)	15 dB (simulated)	Low; numerical validation only	Early concept of active cancellation	Cugnet et al. [99]
Corrective Voltages—Experimental CW	6–10 dB	Low; validated on PZT27 prototypes	Robust to fabrication defects, improved directivity	Bybi et al. [100]
Corrective Voltages—Transient Regime	up to 30 dB	High; FFT-based signal computation	Improved beam directivity	Bybi et al. [101]
Adaptive filtering (NLMS, LMS)	88.9–98.5% noise elimination	Moderate; requires adaptive filtering and band-split processing	Clear imaging optimization, robust to propagation	Lu et al. [102]
Bias-switching in ferroelectric arrays	6.25–7.25 dB	Moderate; uses orthogonal biasing and voltage control	Reduced electrical crosstalk, enhanced dynamic displacement	Park et al. [59]
Neighbor excitation (Golay-coded or opposing signals)	6–9 dB	Low to moderate; waveform shaping	Reduced arrival time fluctuation, improved waveform similarity	Liu et al., Kargary et al., Tong et al. [103,104,105]

**Table 5:** Comprehensive summary of crosstalk mitigation techniques in ultrasound arrays.

Authors	Year	Technology	Crosstalk Mitigation Method/Result
CMUT Arrays			
Voelz et al. [144]	2024	CMUT	Reception CMUT + preamplifier; intrinsic mechanical isolation.
Brock-Fisher [145]	2020	CMUT	ASIC phase compensation in hexagonal array; optimized synchronization.
Hossack and Wojcik [12]	2005	MEMS (Si)	Low-cost transmit/receive structure; improved resolution.
Bayram and Khuri-Yakub [146]	2004	CMUT	Membrane between adjacent elements to reduce coupling.
Zhou and Hossack [14]	2007	CMUT	Transfer matrix + programmable waveforms; −25 to −25.5 dB.
Bayram, Kupnik et al. [49]	2007	CMUT	Periodic membranes to create acoustic band gaps; up to −39 dB.
Hajati et al. [20]	2016	CMUT	Ground plane coupled to substrate; capacitive coupling reduced.
Yongrae Roh et al. [96]	2004	CMUT + Piezo	Structural trenches with polymer walls; 2D FEA validated.
Thomas Lehrmann et al. [147]	2014	CMUT	Row–column addressing architecture.
PMUT Arrays			
Joshi et al. [132]	2023	PMUT	Polyimide/Si rigid PMUTs + physical isolation; ≈1% crosstalk.
Leming He et al. [148]	2017	PMUT (7 × 7)	3D FEM + dual active layers for performance boost.
Xu et al. [67]	2019	PMUT	Resonant cavity design: +259% sensitivity, reduced crosstalk.
Omer M. et al. [57]	2023	PMUT (4 × 4)	Cavity decoupling via FEM and experiments.
Bulk Piezoelectric Arrays (incl. PZT, PZ27)			
Cheng et al. [17]	2022	PZT linear	PZT/epoxy with kerf optimization (PZFlex); −24.1 dB.
Bybi et al. [24]	2020	PZ27	FEM under different electrical boundary conditions; active cancellation.
Lynnworth [136]	1995	Generic piezo	Acoustic/time isolation and packaging; effective separation.
Oliver and Walters [149]	2005	Piezoelectric	Anodic/electrochemical etching for electrical isolation.
S. Zhou et al. [98]	2003	PZT-5H linear	FEM-based corrective tensions on adjacent elements.
Mo et al. [19]	2002	PVDF	Micromachined diaphragm (SW/OW/DW); SW offers low crosstalk.
Cugnet et al. [99]	2002	PZT-5H linear	Transfer function-based excitations for pressure cancellation.
Bybi et al. [100]	2013	Piezoelectric	Corrective voltages on neighbors (FEM-validated).
Bybi et al. [101]	2014	PZ27 linear	Electrical model using motional currents.
Roh and Kim [15]	2002	Convex Piezo	Polymer walls + kerf variation; shape more effective.
Reskal et al. [4]	2023	PZ27	SU8-Epoxy filler + layers; sensitivity/isolation trade-off.
1–3 Piezocomposite Arrays			
Démoré et al. [150]	2007	1–3 Composite	Oblique electrodes reduce periodicity and spurious modes.
Chang Yang et al. [16]	2012	1–3 Composite	Pseudo-random pillar layout to reduce coupling.
Hybrid/Phononic/Other Advanced Structures			
Valappil et al. [66]	2023	Hybrid (PnC)	3D phononic waveguide; 40 dB crosstalk reduction.
Fei et al. [151]	2021	Phononic Piezo plate	Passive isolation via shunts and bandgaps.
Signal Processing/Sparse/Imaging Techniques			
Park et al. [59]	2023	CDMA multi-array	Phase-based demodulator; improved multi-sensor rejection.
Guo et al. [152]	2021	Imaging (TFM)	Half-Matrix Focusing (HFM); faster with less crosstalk.
Ramalli et al. [13]	2019	Sparse array	Topology optimization; sidelobe and crosstalk reduction.
Celmer et al. [31]	2016	Standard ultrasound	FEM with PCB/structural modifications.
Opielinski et al. [26]	2014	Ring array	Crosstalk path/source identification.
Wu Jiahe [153]	2016	Microstrip	Slotted lines + resistors; EM coupling inhibition.
Guo et al. [154]	2004	Flexible ultrasound	Ground traces between lines; reduced electric crosstalk.

## Data Availability

This review is based exclusively on previously published studies. No new datasets were created or analyzed, and therefore data sharing does not apply.
